# Respiratory mortality of childhood, adolescent and young adult cancer survivors

**DOI:** 10.1136/thoraxjnl-2017-210683

**Published:** 2018-05-10

**Authors:** Miranda M Fidler, Raoul C Reulen, Chloe J Bright, Katherine E Henson, Julie S Kelly, Meriel Jenney, Antony Ng, Jeremy Whelan, David L Winter, Clare Frobisher, Michael M Hawkins

**Affiliations:** 1 Centre for Childhood Cancer Survivor Studies, Institute of Applied Health Research, University of Birmingham, Birmingham, UK; 2 Section of Cancer Surveillance, International Agency for Research on Cancer, Lyon, France; 3 Clinical Trial Service Unite, University of Oxford, Oxford, UK; 4 Department of Paediatric Oncology, Children’s Hospital for Wales, Cardiff, UK; 5 Department of Paediatric Oncology, Bristol Royal Hospital for Children, Bristol, UK; 6 Department of Oncology, University College London Hospitals NHS Foundation Trust, London, UK

**Keywords:** clinical epidemiology, copd epidemiology, paediatric interstitial lung disease, pneumonia

## Abstract

**Background:**

Exposure to radiation and/or chemotherapy during cancer treatment can compromise respiratory function. We investigated the risk of long-term respiratory mortality among 5-year cancer survivors diagnosed before age 40 years using the British Childhood Cancer Survivor Study (BCCSS) and Teenage and Young Adult Cancer Survivor Study (TYACSS).

**Methods:**

The BCCSS comprises 34 489 cancer survivors diagnosed before 15 years from 1940 to 2006 in Great Britain. The TYACSS includes 200 945 cancer survivors diagnosed between 15 years and 39 years from 1971 to 2006 in England and Wales. Standardised mortality ratios and absolute excess risks were used.

**Findings:**

Overall, 164 and 1079 respiratory deaths were observed in the BCCSS and TYACSS cohorts respectively, which was 6.8 (95% CI 5.8 to 7.9) and 1.7 (95% CI 1.6 to 1.8) times that expected, but the risks varied substantially by type of respiratory death. Greatest excess numbers of deaths were experienced after central nervous system (CNS) tumours in the BCCSS and after lung cancer, leukaemia, head and neck cancer and CNS tumours in the TYACSS. The excess number of respiratory deaths increased with increasing attained age, with seven (95% CI 2.4 to 11.3) excess deaths observed among those aged 50+ years in the BCCSS and three (95% CI 1.4 to 4.2) excess deaths observed among those aged 60+ years in the TYACSS. It was reassuring to see a decline in the excess number of respiratory deaths among those diagnosed more recently in both cohorts.

**Conclusions:**

Prior to this study, there was almost nothing known about the risks of respiratory death after cancer diagnosed in young adulthood, and this study addresses this gap. These new findings will be useful for both survivors and those involved in their clinical management and follow-up.

**Key messages**

**What is the key question?**

- What are the long-term risks of respiratory-specific mortality among 5-year survivors of childhood, adolescent and young adult cancer?

**What is the bottom line?**

- This large-scale population-based study provides evidence that such survivors are at an increased risk of respiratory death, with appreciable excesses observed among survivors of specific cancers and survivors aged over 50 years, although there is evidence of a reduction in the excess number of respiratory deaths among individuals diagnosed recently.

**Why read on?**

- This article substantially advances the knowledge on respiratory mortality among childhood, adolescent and young adult cancer survivors by providing the most comprehensive analysis to date on respiratory mortality, which is useful for both survivors and those involved in their clinical follow-up.

## Introduction

Survival after childhood, adolescent and young adult cancer has improved substantially over recent decades, with approximately 80% of those diagnosed now surviving at least 5 years.^1 2^ However, the curative treatments used to achieve such outcomes are associated with adverse health effects. One such adverse health effect concerns respiratory diseases (International Classification of Diseases (ICD) 10th revision: J00–J99) which, following circulatory diseases (ICD-10: I00–I99), account for the most non-neoplastic deaths among childhood cancer survivors.^3^ Previous research indicates that direct irradiation to the chest and lungs can cause developmental abnormalities of the thoracic cage, short-term treatment-induced lung disease presenting as pneumonitis or interstitial lung injury, and long-term damage manifesting as fibrosis.^4^ Furthermore, a number of chemotherapy drugs have been associated with respiratory late effects.^4 5^ Such respiratory morbidities can impact the quality of life of survivors^5^ and lead to premature death. As the number of childhood, adolescent and young adult cancer survivors is growing, with all having a substantial proportion of their expected lifespan remaining, it is crucial to investigate the risk of respiratory late effects in this population of survivors in order to identify vulnerable subgroups who may require closer clinical follow-up or intervention, as well as to improve our understanding of the pathogenesis of respiratory complications so that such late effects can be prevented in the future.

We assessed the long-term risks of respiratory-specific mortality among 5-year survivors of cancer diagnosed before the age of 40 years using the British Childhood Cancer Survivor Study (BCCSS) and Teenage and Young Adult Cancer Survivor Study (TYACSS). With over 235 000 survivors, more than 1200 observed respiratory deaths, and nearly 3.5 million person-years available, this study is by far the largest ever assembled to assess long-term respiratory mortality in survivors of childhood, adolescent and young adult cancers. The findings presented provide evidence for respiratory mortality risks overall, as well as the risks of specific types of respiratory death. The evidence produced will be useful for both survivors and those involved in clinical management and follow-up of survivors.

## Methods

### Study populations

This study uses two of the largest population-based cohorts of childhood, adolescent and young adult cancer survivors: the BCCSS^6^ and TYACSS.^7^ The BCCSS comprises 34 489 5-year survivors of childhood cancer diagnosed under the age of 15 years in Great Britain from 1940 to 2006. The cohort was identified using the National Registry of Childhood Tumors,^8^ and all first primary neoplasms (FPN) were classified using the International Classification of Childhood Cancer (online supplementary etable 1).^9^ The TYACSS includes 200 945 5-year survivors of adolescent and young adult cancers diagnosed between 1971 and 2006, in England and Wales, when aged between 15 years and 39 years. The cohort was established in collaboration with the Office for National Statistics and Welsh Cancer Registry. Adolescent and young adult FPNs were classified using the categories proposed by Birch *et al*, which were then slightly modified to produce finer groupings (online supplementary etable 2).^10 11^

10.1136/thoraxjnl-2017-210683.supp1 Supplementary file 1

### Death ascertainment

The BCCSS and TYACSS cohorts were linked to the National Death Registration systems in order to ascertain each survivor’s vital and emigration status. This linkage provided the death certificate and underlying cause of death, as coded using the relevant ICD at time of death. Respiratory deaths were determined by using the ‘diseases of the respiratory system’ chapters of the relevant ICD editions. ICD codes were further subcategorised into the following clinically relevant groups for analysis: pneumonia, chronic lower respiratory disease (excluding asthma), fibrosis, pneumonitis and other respiratory deaths (online supplementary etable 3). Follow-up for respiratory mortality commenced at 5-year survival and continued until the first occurrence of emigration, death or 28 February 2014.

### Statistical analyses

To investigate the risk of respiratory mortality among survivors compared with that expected from the general population, standardised mortality ratios (SMRs) and absolute excess risks (AERs) were calculated using standard cohort techniques.^12^ The SMR was defined as the ratio of the observed over the expected number of respiratory deaths. The AER was defined as the observed minus the expected number of respiratory deaths divided by person-years at risk multiplied by 10 000; when reporting AERs, we often simply provide the excess, or extra, number of deaths without specifying the 10 000 person-years denominator. Expected numbers were calculated by multiplying the person-years at risk for each sex-specific, age-specific (5-year bands) and calendar year-specific (1-year bands) stratum by the corresponding respiratory mortality rate for the population of England and Wales and then summing across the strata.^13^ Likelihood ratio tests comparing multivariate Poisson regression models adjusting for the simultaneous effects of sex, FPN type, age at cancer diagnosis, treatment era and attained age were used to test for significant heterogeneity or a significant linear trend in the adjusted SMRs and AERs for each explanatory factor. In reporting, we focus on AERs because of their direct and clear interpretation as the excess number of deaths beyond that expected, indicating extent of adverse public health impact. All analyses were completed using Stata Corp, 2013 V.13.1, where the criterion for statistical significance was a two-sided P<0.05.

### Role of the funding source

The funder had no role in this study. The lead and corresponding authors had full access to all the data in the study and had final responsibility of the decision to submit for publication.

## Results

### British Childhood Cancer Survivor Study

#### All respiratory deaths

The BCCSS cohort was followed up for a total of 620 758 person-years from 5-year survival. The mean follow-up from FPN diagnosis was 23.0 years (range: 5.0–73.7) and mean attained age at the end of current follow-up was 29.6 years (range: 5.5–85.6). Of the 4475 deaths observed, 164 (3.7%) were due to respiratory causes (table 1). Over half of the respiratory deaths were observed in survivors of central nervous system (CNS) tumours, including CNS primitive neuroectodermal tumours (PNET). However, when the proportion of deaths attributable to respiratory causes was assessed, germ cell tumour survivors had the highest percentage at nearly 11%.

**Table 1:** Study characteristics of the British Childhood Cancer Survivor Study

Patient characteristic	Respiratory death	%	Other death	%	Total
Overall	164	3.7	4311	96.3	4475
Sex					
Male	98	3.7	2531	96.3	2629
Female	66	3.6	1780	96.3	1848
First primary neoplasm type					
CNS tumour (excluding PNET)	68	5.3	1223	94.7	1291
CNS PNET	15	4.4	326	95.6	341
Leukaemia (excluding AML)	23	2.1	1080	97.9	1103
AML	3	3.7	79	96.3	82
Hodgkin’s lymphoma	7	2.1	324	97.9	331
Non-Hodgkin’s lymphoma	5	3.8	126	96.2	131
Neuroblastoma	6	4.2	138	95.8	144
Non-heritable retinoblastoma	1	3.2	30	96.8	31
Heritable retinoblastoma	4	2.9	134	97.1	138
Wilms	7	3.8	177	96.2	184
Bone sarcoma	1	0.5	197	99.5	198
Soft tissue sarcoma	11	4.3	242	95.7	253
Germ cell tumours	8	10.7	67	89.3	75
Other	5	2.9	168	97.1	173
Age at diagnosis (years)					
Mean	7.3		7.3		7.3
0–4	64	3.9	1597	96.1	1661
5–9	43	3.2	1309	96.8	1352
10–14	57	3.9	1405	96.1	1462
Treatment era					
1940–1969	61	4.6	1268	95.4	1329
1970–1979	48	3.8	1199	96.2	1247
1980–1989	36	3.8	905	96.2	941
1990–2006	19	2.0	939	98.0	958
Years from diagnosis					
Mean	24.7		16.7		17.0
5–9	36	1.7	2022	98.3	2058
10–19	33	3.1	1028	96.9	1061
20–29	33	6.0	515	94.0	548
30–39	32	7.6	390	92.4	422
40–49	21	7.6	256	92.4	277
50–59	8	7.7	96	92.3	104
60+	1	5.9	16	94.1	17
Attained age at exit (years)					
Mean	32.0		24.0		24.3
5–9	7	1.7	409	98.3	416
10–19	37	2.0	1797	98.0	1834
20–29	35	3.5	970	96.5	1005
30–39	34	6.3	502	93.7	536
40–49	27	7.3	341	92.7	368
50–59	20	8.4	218	91.6	238
60+	4	4.4	86	95.6	90

AML, acute myeloid leukaemia; CNS, central nervous system; PNET, primitive neuroectodermal tumour.

**Figure 1:**
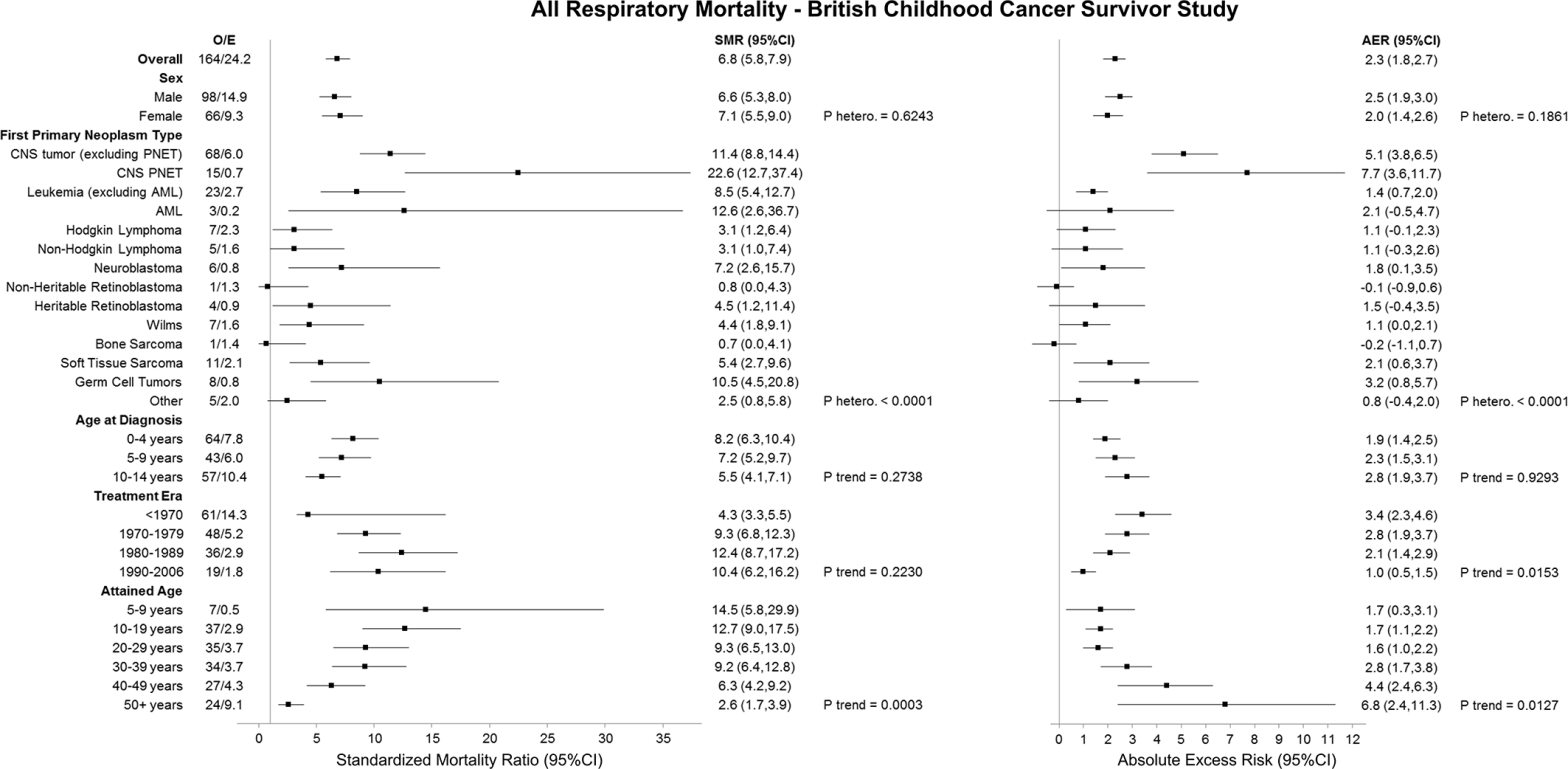
SMRs and AERs per 10 000 person-years for all respiratory mortality in the British Childhood Cancer Survivor Study. P heterogeneity and p trend were calculated by using likelihood ratio tests within multivariable Poisson regression models that adjusted for sex, first primary neoplasm type, age at diagnosis, treatment era and attained age. Strata with less than five observed events should be interpreted with caution. AER, absolute excess risk; AML, acute myeloid leukaemia; CNS, central nervous system; E, expected; O, observed; PNETs, primitive neuroectodermal tumour; SMR, standardised mortality ratio.

Survivors of childhood cancer were 6.8 times (95% CI 5.8 to 7.9) more at risk of respiratory death than expected in the general population; this equated to 2.3 (95% CI 1.8 to 2.7) excess respiratory deaths (figure 1). Survivors of each FPN type with at least 5 observed deaths were found to be significantly more at risk of respiratory death than expected; the excess number of respiratory deaths exceeded 5 among survivors of CNS PNET and CNS tumours (excluding PNET) at 7.7 (95% CI 3.6 to 11.7) and 5.1 (95% CI 3.8 to 6.5), respectively. As attained age increased, the AERs increased (adjusted p trend=0.0127); at 5–19 years age, the AER was 1.7 (95% CI 0.3 to 3.1), while beyond 50 years age the AER was 6.8 (95% CI 2.4 to 11.3). Among those treated more recently, the number of excess respiratory deaths declined (adjusted p trend=0.0153); survivors diagnosed before 1970 and between 1990 and 2006 experienced 3.4 (95% CI 2.3 to 4.6) and 1.0 (95% CI 0.5 to 1.5) excess respiratory deaths, respectively.

Of the 164 respiratory deaths observed, there were 77 pneumonia, 18 pneumonitis, 15 fibrosis, 11 chronic lower respiratory disease and 43 other respiratory deaths (online supplementary etable 4). The corresponding SMRs were 8.2 (95% CI 6.5 to 10.2), 16.9 (95% CI 10.0 to 26.8), 13.8 (95% CI 7.7 to 22.8), 1.8 (95% CI 0.9 to 3.2) and 6.5 (95% CI 4.7 to 8.8), respectively, and the corresponding AERs were 1.1 (95% CI 0.8 to 1.4), 0.3 (95% CI 0.1 to 0.4), 0.2 (95% CI 0.1 to 0.3), 0.1 (95% CI 0.0 to 0.2) and 0.6 (95% CI 0.4 to 0.8). As nearly half of the respiratory deaths observed were due to pneumonia, further analyses were only undertaken for this specific group of deaths. However, it is worth noting that 13/18 (72.2%) of the pneumonitis deaths occurred in CNS tumours (excluding PNET) survivors, which equated to a 53.8-fold (95% CI 28.6 to 92.0) increased risk compared with that expected.

### Pneumonia deaths

**Figure 2:**
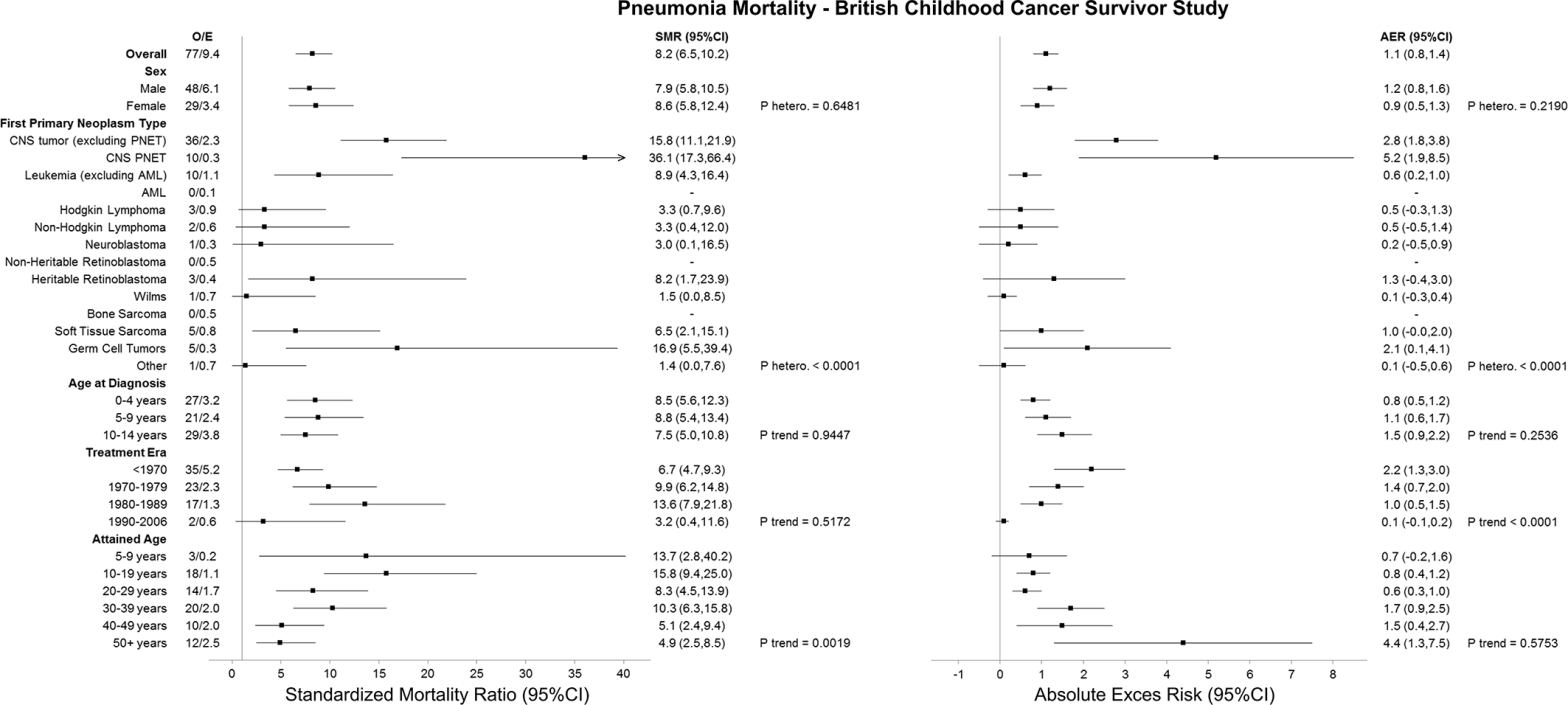
SMRs and AERs per 10 000 person-years for pneumonia mortality in the British Childhood Cancer Survivor Study. P heterogeneity and p trend were calculated by using likelihood ratio tests within multivariable Poisson regression models that adjusted for sex, first primary neoplasm type, age at diagnosis, treatment era and attained age. Strata with less than five observed events should be interpreted with caution. AER, absolute excess risk; AML, acute myeloid leukaemia; CNS, central nervous system; E, expected; O, observed; PNETs, primitive neuroectodermal tumours; SMR, standardised mortality ratio.

Survivors of CNS PNET, CNS tumours (excluding PNET) and germ cell tumours experienced the greatest excess numbers of pneumonia deaths at 5.2 (95% CI 1.9 to 8.5), 2.8 (95% CI 1.8 to 3.8) and 2.1 (95% CI 0.1 to 4.1), respectively (figure 2). With more recent treatment era, there was a decline in the excess number of pneumonia deaths observed (adjusted p trend <0.0001), where the AER decreased from 2.2 (95% CI 1.3 to 3.0) to 0.1 (95% CI −0.1 to 0.2) among those diagnosed before 1970 and between 1990 and 2006, respectively.

### Teenage and Young Adult Cancer Survivor Study

#### All respiratory deaths

From 5-year survival, the TYACSS cohort was followed up for a total of 2 867 878 person-years. The mean follow-up from FPN diagnosis was 19.3 years (range: 5.0–43.2) and mean attained age at exit was 51.0 years (range: 20.3–82.7). Overall, 34 180 (17.0%) adolescent and young adult survivors had died; of these, 1079 (3.2%) were due to respiratory causes (table 2). The proportion of deaths attributable to respiratory causes was highest among lung cancer survivors at 10%, followed by head and neck cancer survivors and cervical cancer survivors at 6% each.

**Table 2:** Study characteristics of the teenage and young adult cancer survivor study

Patient characteristic	Respiratory death	%	Other death	%	Total
Overall	1079	3.2	33 101	96.8	34 180
Sex					
Male	479	4.0	11 606	96.1	12 085
Female	600	2.7	21 495	97.3	22 095
First primary neoplasm type					
Breast	135	1.3	10 474	98.7	10 609
Testicular	98	4.9	1909	95.1	2007
Cervical	152	5.8	2490	94.3	2642
Melanoma	25	1.1	2293	98.9	2318
CNS tumour	141	3.4	3958	96.6	4099
Hodgkin’s lymphoma	134	4.4	2937	95.6	3071
Non-Hodgkin’s lymphoma	72	4.2	1647	95.8	1719
Thyroid	13	3.1	402	96.9	415
Gastrointestinal	47	3.4	1346	96.6	1393
Soft tissue sarcoma	30	3.4	863	96.6	893
Ovary	22	3.1	695	96.9	717
Bladder	30	5.2	552	94.9	582
Other genitourinary	35	4.1	819	95.9	854
Head and neck	42	6.1	648	93.9	690
Leukaemia (excluding AML)	25	3.3	743	96.7	768
Other	26	4.9	501	95.1	527
Bone tumour	11	3.0	361	97.0	372
AML	12	5.5	205	94.5	217
Lung	29	10.1	258	89.9	287
Age at diagnosis (years)					
Mean	33.2		33.1		33.1
15–19	53	3.8	1360	96.3	1413
20–24	83	3.4	2326	96.6	2409
25–29	141	3.0	4584	97.0	4725
30–34	257	2.8	8924	97.2	9181
35–39	545	3.3	15 907	96.7	16 452
Treatment era					
1971–1979	459	4.9	8972	95.1	9431
1980–1989	412	3.3	11 908	96.7	12 320
1990–1990	169	1.9	8811	98.1	8980
2000–2006	39	1.1	3410	98.9	3449
Years from diagnosis					
Mean	22.0		14.5		14.8
5–9	159	1.1	14 235	98.9	14 394
10–19	280	2.6	10 505	97.4	10 785
20–29	384	6.3	5722	93.7	6106
30–39	245	8.9	2500	91.1	2745
40+	11	7.3	139	92.7	150
Attained age at exit (years)					
Mean	55.2		47.7		47.9
20–29	20	1.6	1252	98.4	1272
30–39	105	1.8	5731	98.2	5836
40–49	229	1.6	14 420	98.4	14 649
50–59	317	4.2	7177	95.8	7497
60–69	307	7.7	3661	92.3	3968
70+	101	10.5	858	89.5	959

AML, acute myeloid leukaemia; CNS, central nervous system.

**Figure 3:**
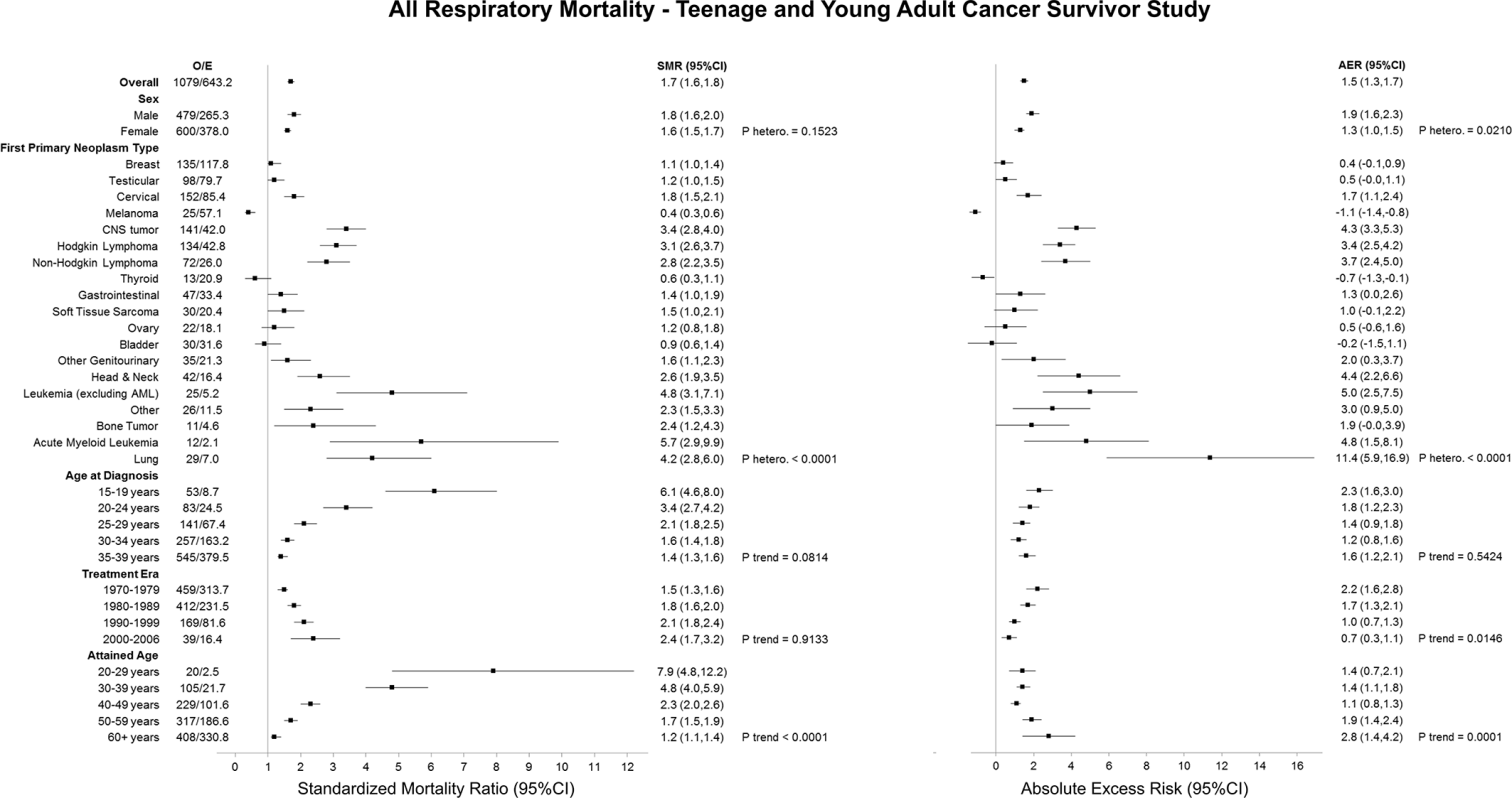
SMRs and AERs per 10 000 person-years for all respiratory mortality in the Teenage and Young Adult Cancer Survivor Study. P heterogeneity and p trend were calculated by using likelihood ratio tests within multivariable Poisson regression models that adjusted for sex, first primary neoplasm type, age at diagnosis, treatment era and attained age. Strata with less than five observed events should be interpreted with caution. AER, absolute excess risk; AML, acute myeloid leukaemia; CNS, central nervous system; E, expected; O, observed; PNETs, primitive neuroectodermal tumour; SMR, standardised mortality ratio.

Overall, adolescent and young adult survivors were 70% (SMR: 1.7; 95% CI 1.6 to 1.8) more likely to die from a respiratory cause than expected from the general population, which equated to 1.5 (95% CI 1.3 to 1.7) excess respiratory deaths (figure 3). The excess number of respiratory deaths was significantly higher among males compared with females (adjusted p heterogeneity=0.0210). Survivors of lung, leukaemia (excluding acute myeloid leukaemia (AML)), head and neck cancer and CNS tumours experienced the greatest excess number of respiratory deaths at 11.4 (95% CI 5.9 to 16.9), 5.0 (95% CI 2.5 to 7.5), 4.8 (95% CI 1.5 to 8.1), 4.4 (95% CI 2.2 to 6.6) and 4.3 (95% CI 3.3 to 5.3), respectively. As attained age increased. the AERs increased from 1.4 (95% CI 0.7 to 2.1) among those aged 20–29 years to 2.8 (95% CI 1.4 to 4.2) among those aged 60+ years (adjusted p trend=0.0001). A decline in the excess number of respiratory deaths with more recent treatment era was observed (adjusted p trend=0.0146), where the AER decreased from 2.2 (95% CI 1.6 to 2.8) to 0.7 (95% CI 0.3 to 1.1) in those treated in the 1970s and 2000s, respectively.

**Figure 4:**
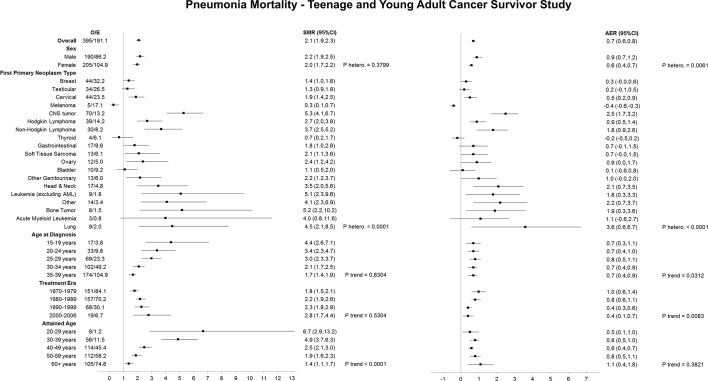
SMRs and AERs per 10 000 person-years for pneumonia mortality in the Teenage and Young Adult Cancer Survivor Study. P heterogeneity and p trend were calculated by using likelihood ratio tests within multivariable Poisson regression models that adjusted for sex, first primary neoplasm type, age at diagnosis, treatment era and attained age. Strata with less than five observed events should be interpreted with caution. AER, absolute excess risk; AML, acute myeloid leukaemia; CNS, central nervous system; E, expected; O, observed; PNETs, primitive neuroectodermal tumour; SMR, standardised mortality ratio

When the 1079 respiratory deaths were assessed by specific respiratory causes of death, there were 395 pneumonia, 393 chronic lower respiratory disease, 74 fibrosis, 49 pneumonitis and 168 other respiratory deaths (figure 4, table 3). Although adolescent and young adult survivors were at a statistically significant increased risk of death from each specific type of respiratory death, the SMR was greatest for pneumonitis and pneumonia deaths at 2.2 (95% CI 1.6 to 2.9) and 2.1 (95% CI 1.9 to 2.3), respectively.

**Table 3:** SMRs and AERs per 10 000 person-years for chronic lower respiratory mortality, fibrosis mortality, pneumonitis mortality and other respiratory mortality by potential explanatory factors, for the Teenage and Young Adult Cancer Survivor Study^†^

	Chronic lower respiratory disease			Fibrosis			Pneumonitis			Other respiratory		
	O/E	SMR (95% CI)	AER (95% CI)	O/E	SMR (95% CI)	AER (95% CI)	O/E	SMR (95% CI)	AER (95% CI)	O/E	SMR (95% CI)	AER (95% CI)
Overall	393/295.0	1.3 (1.2 to 1.5)	0.3 (0.2 to 0.5)	74/42.7	1.7 (1.4 to 2.2)	0.1 (0.1 to 0.2)	49/22.5	2.2 (1.6 to 2.9)	0.1 (0.0 to 0.1)	168/91.9	1.8 (1.6 to 2.1)	0.3 (0.2 to 0.4)
Sex												
Male	146/112.1	1.3 (1.1 to 1.5)	0.3 (0.1 to 0.5)	29/20.7	1.4 (0.9 to 2.0)	0.1 (−0.0 to 0.2)	28/10.3	2.7 (1.8 to 3.9)	0.2 (0.1 to 0.3)	86/36.1	2.4 (1.9 to 2.9)	0.5 (0.3 to 0.6)
Female	247/183.0	1.4 (1.2 to 1.5)	0.4 (0.2 to 0.5)	45/22.0	2.0 (1.5 to 2.7)	0.1 (0.1 to 0.2)	21/12.2	1.7 (1.1 to 2.6)	0.0 (−0.0 to 0.1)	82/55.8	1.5 (1.2 to 1.8)	0.1 (0.0 to 0.2)
P hetero*		0.2252	0.3215		0.0007	0.0040		0.7433	0.2161		0.2266	0.1113
First primary neoplasm type												
Breast	56/58.4	1.0 (0.7 to 1.2)	−0.1 (−0.4 to 0.3)	8/7.0	1.2 (0.5 to 2.3)	0.0 (−0.1 to 0.1)	4/3.6	1.1 (0.3 to 2.8)	0.0 (−0.1 to 0.1)	23/16.6	1.4 (0.9 to 2.1)	0.1 (−0.1 to 0.4)
Testicular	37/32.7	1.1 (0.8 to 1.6)	0.1 (−0.2 to 0.5)	9/6.1	1.5 (0.7 to 2.8)	0.1 (−0.1 to 0.2)	3/3.2	0.9 (0.2 to 2.7)	−0.0 (−0.1 to 0.1)	15/11.2	1.3 (0.8 to 2.2)	0.1 (−0.1 to 0.3)
Cervical	87/41.6	2.1 (1.7 to 2.6)	1.2 (0.7 to 1.7)	7/5.0	1.4 (0.6 to 2.9)	0.1 (−0.1 to 0.2)	2/2.8	0.7 (0.1 to 2.6)	−0.0 (−0.1 to 0.1)	12/12.5	1.0 (0.5 to 1.7)	−0.0 (−0.2 to 0.2)
Melanoma	13/25.7	0.5 (0.3 to 0.9)	−0.4 (−0.7 to −0.2)	2/3.7	0.5 (0.1 to 2.0)	−0.1 (−0.2 to 0.0)	2/2.1	0.9 (0.1 to 3.4)	−0.0 (−0.1 to 0.1)	3/8.5	0.4 (0.1 to 1.0)	−0.2 (−0.3 to -0.1)
CNS tumour	21/18.1	1.2 (0.7 to 1.8)	0.1 (−0.3 to 0.5)	3/2.8	1.1 (0.2 to 3.1)	0.0 (−0.1 to 0.2)	20/1.6	12.8 (7.8 to 19.8)	0.8 (0.4 to 1.2)	27/6.3	4.3 (2.8 to 6.2)	0.9 (0.5 to 1.3)
Hodgkin’s lymphoma	40/17.4	2.3 (1.6 to 3.1)	0.8 (0.4 to 1.3)	19/2.8	6.7 (4.0 to 10.4)	0.6 (0.3 to 0.9)	3/1.6	1.8 (0.4 to 5.4)	0.1 (−0.1 to 0.2)	33/6.7	4.9 (3.4 to 6.9)	1.0 (0.6 to 1.4)
Non-Hodgkin’s lymphoma	15/11.3	1.3 (0.7 to 2.2)	0.3 (−0.3 to 0.9)	4/1.8	2.2 (0.6 to 5.5)	0.2 (−0.1 to 0.5)	3/1.0	3.1 (0.6 to 9.0)	0.2 (−0.1 to 0.4)	20/3.7	5.3 (3.3 to 8.2)	1.3 (0.6 to 2.0)
Thyroid	6/9.6	0.6 (0.2 to 1.4)	−0.3 (−0.7 to 0.1)	0/1.3	–	–	0/0.7	–	–	3/3.1	1.0 (0.2 to 2.8)	−0.0 (−0.3 to 0.3)
Gastrointestinal	20/16.0	1.3 (0.8 to 1.9)	0.4 (−0.5 to 1.2)	3/2.4	1.2 (0.3 to 3.6)	0.1 (−0.3 to 0.4)	0/1.1	–	–	7/4.3	1.6 (0.7 to 3.4)	0.3 (−0.2 to 0.8)
Soft tissue sarcoma	13/9.2	1.4 (0.8 to 2.4)	0.4 (−0.4 to 1.2)	1/1.4	0.7 (0.0 to 4.0)	−0.0 (−0.3 to 0.2)	1/0.7	1.4 (0.0 to 7.7)	0.0 (−0.2 to 0.2)	2/2.9	0.7 (0.1 to 2.5)	−0.1 (−0.4 to 0.2)
Ovary	7/8.9	0.8 (0.3 to 1.6)	−0.2 (−0.9 to 0.4)	0/1.1	–	–	0/0.6	–	–	3/2.6	1.2 (0.2 to 3.4)	0.0 (−0.4 to 0.5)
Bladder	16/15.0	1.1 (0.6 to 1.7)	0.1 (−0.8 to 1.1)	3/2.6	1.2 (0.2 to 3.4)	0.1 (−0.4 to 0.5)	0/1.1	–	–	1/3.8	0.3 (0.0 to 1.5)	−0.3 (−0.6 to -0.1)
Other genitourinary	14/10.4	1.3 (0.7 to 2.3)	0.5 (−0.5 to 1.6)	4/1.4	2.8 (0.8 to 7.1)	0.4 (−0.2 to 0.9)	1/0.7	1.5 (0.0 to 8.1)	0.0 (−0.2 to 0.3)	3/2.8	1.1 (0.2 to 3.1)	0.0 (−0.5 to 0.5)
Head and neck	16/7.6	2.1 (1.2 to 3.4)	1.5 (0.1 to 2.8)	1/1.2	0.8 (0.0 to 4.5)	−0.0 (−0.4 to 0.3)	5/0.6	8.8 (2.9 to 20.6)	0.8 (0.0 to 1.5)	3/2.1	1.4 (0.3 to 4.1)	0.1 (−0.4 to 0.7)
Leukaemia (excluding AML)	6/2.0	3.0 (1.1 to 6.6)	1.0 (−0.2 to 2.2)	4/0.3	12.1 (3.3 to 31.0)	0.9 (−0.1 to 1.9)	3/0.2	13.7 (2.8 to 39.9)	0.7 (−0.2 to 1.6)	3/0.9	3.4 (0.7 to 10.0)	0.5 (−0.3 to 1.4)
Other	8/5.3	1.5 (0.6 to 2.9)	0.5 (−0.6 to 1.7)	1/0.8	1.2 (0.0 to 6.9)	0.0 (−0.4 to 0.4)	1/0.4	2.5 (0.1 to 14.0)	0.1 (−0.3 to 0.5)	2/1.6	1.2 (0.2 to 4.5)	0.1 (−0.5 to 0.6)
Bone tumour	1/1.8	0.6 (0.0 to 3.1)	−0.2 (−0.8 to 0.3)	1/0.3	3.3 (0.1 to 18.3)	0.2 (−0.4 to 0.8)	1/0.2	5.4 (0.1 to 30.0)	0.2 (−0.3 to 0.8)	0/0.8	–	–
AML	1/0.7	1.4 (0.0 to 7.6)	0.1 (−0.8 to 1.1)	3/0.1	24.1 (5.0 to 70.6)	1.4 (−0.3 to 3.1)	0/0.1	–	–	5/0.4	12.6 (4.1 to 29.3)	2.2 (0.1 to 4.4)
Lung	16/3.3	4.8 (2.7 to 7.8)	6.6 (2.5 to 10.6)	1/0.5	1.8 (0.0 to 10.2)	0.2 (−0.8 to 1.3)	0/0.2	–	–	3/0.9	3.5 (0.7 to 10.1)	1.1 (−0.7 to 2.9)
P hetero*		<0.0001	<0.0001		<0.0001	0.0001		<0.0001	<0.0001		<0.0001	<0.0001
Age at diagnosis (years)												
15–19	9/1.6	5.6 (2.6 to 10.6)	0.4 (0.1 to 0.7)	8/0.4	21.5 (9.3 to 42.3)	0.4 (0.1 to 0.7)	4/0.5	8.4 (2.3 to 21.4)	0.2 (−0.0 to 0.4)	15/2.4	6.3 (3.5 to 10.4)	0.7 (0.3 to 1.0)
20–24	18/6.9	2.6 (1.5 to 4.1)	0.3 (0.1 to 0.6)	9/1.3	7.1 (3.2 to 13.4)	0.2 (0.1 to 0.4)	7/1.2	5.9 (2.4 to 12.1)	0.2 (0.0 to 0.3)	16/5.3	3.0 (1.7 to 4.9)	0.3 (0.1 to 0.6)
25–29	30/25.3	1.2 (0.8 to 1.7)	0.1 (−0.1 to 0.3)	7/4.0	1.7 (0.7 to 3.6)	0.1 (−0.0 to 0.2)	8/2.8	2.8 (1.2 to 5.5)	0.1 (−0.0 to 0.2)	27/11.8	2.3 (1.5 to 3.3)	0.3 (0.1 to 0.5)
30–34	85/73.2	1.2 (0.9 to 1.4)	0.2 (−0.1 to 0.4)	21/10.8	1.9 (1.2 to 3.0)	0.1 (0.0 to 0.2)	10/6.0	1.7 (0.8 to 3.1)	0.1 (−0.0 to 0.1)	39/24.0	1.6 (1.2 to 2.2)	0.2 (0.0 to 0.4)
35–39	251/188.0	1.3 (1.2 to 1.5)	0.6 (0.3 to 0.9)	29/26.2	1.1 (0.7 to 1.6)	0.0 (−0.1 to 0.1)	20/12.0	1.7 (1.0 to 2.6)	0.1 (−0.0 to 0.2)	71/48.3	1.5 (1.1 to 1.9)	0.2 (0.1 to 0.4)
P trend*		0.8584	0.3593		0.3378	0.1290		0.4927	0.1572		0.0340	0.2243
Treatment era												
1970–1979	210/160.9	1.3 (1.1 to 1.5)	0.7 (0.3 to 1.2)	28/22.5	1.2 (0.8 to 1.8)	0.1 (−0.1 to 0.2)	23/8.6	2.7 (1.7 to 4.0)	0.2 (0.1 to 0.4)	47/37.6	1.3 (0.9 to 1.7)	0.1 (−0.1 to 0.3)
1980–1989	146/105.4	1.4 (1.2 to 1.6)	0.4 (0.2 to 0.6)	27/14.7	1.8 (1.2 to 2.7)	0.1 (0.0 to 0.2)	14/8.2	1.7 (0.9 to 2.9)	0.1 (−0.0 to 0.1)	68/33.1	2.1 (1.6 to 2.6)	0.3 (0.2 to 0.5)
1990–1999	33/25.4	1.3 (0.9 to 1.8)	0.1 (−0.0 to 0.2)	17/4.7	3.6 (2.1 to 5.8)	0.1 (0.0 to 0.2)	9/4.5	2.0 (0.9 to 3.8)	0.1 (−0.0 to 0.1)	42/16.9	2.5 (1.8 to 3.4)	0.3 (0.1 to 0.4)
2000–2006	4/3.3	1.2 (0.3 to 3.1)	0.0 (−0.1 to 0.2)	2/0.9	2.3 (0.3 to 8.5)	0.0 (−0.1 to 0.1)	3/1.2	2.6 (0.5 to 7.5)	0.1 (−0.1 to 0.2)	11/4.3	2.5 (1.3 to 4.5)	0.2 (0.0 to 0.4)
P trend*		0.2334	0.0024		0.6104	0.9954		0.0942	0.5108		0.0066	0.1235
Attained age (years)												
20–29	2/0.1	16.2 (2.0 to 58.7)	0.1 (−0.1 to 0.4)	2/0.1	39.2 (4.7 to 141.5)	0.2 (−0.1 to 0.4)	1/0.1	8.1 (0.2 to 45.3)	0.1 (−0.1 to 0.2)	7/1.0	6.7 (2.7 to 13.8)	0.5 (0.1 to 0.9)
30–39	7/1.7	4.1 (1.7 to 8.5)	0.1 (0.0 to 0.2)	14/0.6	22.4 (12.3 to 37.6)	0.2 (0.1 to 0.4)	7/1.0	6.8 (2.7 to 14.1)	0.1 (0.0 to 0.2)	21/6.8	3.1 (1.9 to 4.7)	0.2 (0.1 to 0.4)
40–49	38/21.6	1.8 (1.2 to 2.4)	0.1 (0.0 to 0.2)	13/4.4	2.9 (1.6 to 5.0)	0.1 (0.0 to 0.1)	12/4.5	2.7 (1.4 to 4.7)	0.1 (0.0 to 0.1)	52/25.7	2.0 (1.5 to 2.7)	0.2 (0.1 to 0.3)
50–59	122/82.3	1.5 (1.2 to 1.8)	0.6 (0.3 to 0.9)	23/10.9	2.1 (1.3 to 3.2)	0.2 (0.0 to 0.3)	10/6.9	1.5 (0.7 to 2.7)	0.0 (−0.0 to 0.1)	50/28.3	1.8 (1.3 to 2.3)	0.3 (0.1 to 0.5)
60+	224/189.3	1.2 (1.0 to 1.3)	1.3 (0.2 to 2.3)	22/26.7	0.8 (0.5 to 1.2)	−0.2 (−0.5 to 0.2)	19/10.0	1.9 (1.1 to 3.0)	0.3 (0.0 to 0.6)	38/30.1	1.3 (0.9 to 1.7)	0.3 (−0.2 to 0.7)
P trend*		0.0005	<0.0001		<0.0001	0.3255		0.0866	0.0078		0.8761	0.0070

*P heterogeneity and p trend were calculated by using likelihood ratio tests within multivariable Poisson regression models that adjusted for sex, first primary neoplasm type, age at diagnosis, treatment era and attained age.

†Strata with less than five observed events should be interpreted with caution.

AER, absolute excess risk; AML, acute myeloid leukaemia; CNS, central nervous system; E, expected; O, observed; PNETs, primitive neuroectodermal tumour; SMR, standardised mortality ratio.

#### Pneumonia deaths

Male survivors of adolescent and young adult cancers experienced more excess pneumonia mortality compared with females (adjusted p heterogeneity=0.0061) (figure 4). Survivors of lung cancer, CNS tumours and head and neck cancers experienced the greatest excess number of pneumonia deaths at 3.6 (95% CI 0.6 to 6.7), 2.5 (95% CI 1.7 to 3.2) and 2.1 (95% CI 0.7 to 3.5), respectively. The excess number of pneumonia deaths increased as age at diagnosis increased (adjusted p trend=0.0312) and declined among those treated more recently (adjusted p trend=0.0083).

#### Chronic lower respiratory disease deaths

Survivors of lung cancer experienced the greatest excess number of deaths at 6.6 (95% CI 2.5 to 10.6) (table 3). AERs increased with attained age from 0.1 (95% CI −0.1 to 0.4) to 1.3 (95% CI 0.2 to 2.3) among those aged 20–29 years and 60+ years, respectively (adjusted p trend <0.0001). Survivors diagnosed more recently experienced less excess chronic lower respiratory disease deaths (adjusted p trend=0.0024).

### Fibrosis and pneumonitis deaths

With regards to fibrosis death, female survivors were found to experience a greater excess number of deaths than male survivors (adjusted p heterogeneity=0.0040) (table 3). For both fibrosis and pneumonitis deaths, the AERs were consistently less than one after each specific FPN type, except for fibrosis deaths among AML survivors, though only three deaths were observed. Finally, the AER increased with increasing attained age for pneumonitis deaths (adjusted p trend=0.0078).

## Discussion

Compared with the previous literature, our study is by far the largest to assess respiratory mortality among 5-year survivors of childhood, adolescent and young adult cancers, reporting for the first time results for specific types of respiratory death and risks beyond 50 years of age. There have been two previous studies of survivors of cancer diagnosed in individuals aged at least 20 years—one included 11 417 5-year survivors and 29 respiratory deaths,^14^ the other included 1248 5-year survivors and 7 respiratory deaths,^15^ and we included 188 697 5-year survivors and 1079 respiratory deaths. As a result of these large numbers, and our study’s population-based design and long period of follow-up, we provide the most comprehensive investigation of risks and risk factors for respiratory mortality among childhood, adolescent and young adult cancer survivors to date.

Our principal new findings include the following. Among survivors of childhood cancer, the greatest excess numbers of respiratory deaths were experienced after PNETs of the CNS and other CNS tumours at 7.7 and 5.1 extra, respectively. Among survivors of adolescent and young adult cancer, the greatest excess number of respiratory deaths was experienced after lung cancer, leukaemia (excluding AML), AML, head and neck and CNS tumours at 11.4, 5.0, 4.8, 4.4 and 4.3 extra, respectively. The excess number of respiratory deaths increased with increasing attained age, reaching 6.8 excess deaths among those aged 50+ years in the BCCSS, and reaching 2.8 excess deaths among those aged 60+ years in the TYACSS cohort. It was reassuring that the excess number of respiratory deaths declined among those treated more recently in both cohorts, and in each cohort, the AER declined to about one excess respiratory death among those treated most recently.

### Our findings in relation to previous research

In this study, childhood cancer survivors were seven times more at risk of respiratory death than that expected from the general population, while adolescent and young adult cancer survivors were two times more at risk; these findings broadly correspond with previous reports.^14–16^ However, when assessed in more detail, the excess risks for specific types of respiratory death varied considerably. Significant excesses were observed for pneumonia, fibrosis, pneumonitis and other respiratory deaths for childhood cancer survivors, with the SMRs ranging from 7-fold to 17-fold that expected. Similarly, risks varied among adolescent and young adult cancer survivors depending on the specific type of respiratory death, although to a lesser extent than that observed among childhood cancer survivors. The fact that the SMR among childhood cancer survivors was higher than the corresponding SMR in adolescents and young adults for each type of respiratory death assessed could relate to the increased vulnerability of children to radiotherapy, chemotherapy and surgery side effects as the child is still growing and developing,^17^ while adolescents and young adults generally tolerate acute side effects of therapy relatively well.^18^

In regards to risk factors, the greatest excess numbers of respiratory deaths among survivors of childhood cancer were experienced after CNS PNET and other CNS tumours, and after lung cancer, leukaemia (excluding AML), AML, head and neck and CNS tumours among survivors of adolescent and young adult cancer. The large increased risks observed in these specific FPN types could relate to previous cancer treatment.^5 19^ For example, craniospinal irradiation and the use of nitrosureas in CNS survivors can induce pneumonitis and late restrictive lung disease,^20^ while restrictive and obstructive lung disease can develop as a result of the use craniospinal irradiation, total body irradiation or high-dose busulphan in bone marrow transplantation for leukaemia survivors.^19 21^ Other clinical correlations between therapeutic agents and respiratory late effects are as follows: (1) busulfan, carmustine or lomustine and the development of pulmonary fibrosis; (2) bleomycin and the development of interstitial pneumonitis, pulmonary fibrosis or acute respiratory distress syndrome; (3) irradiation to the axilla, chest, extended mantle, mantle, mediastinal, mini-mantle or whole lung, subtotal lymphoid irradiation, total body irradiation or total lymphoid irradiation and the development of pulmonary fibrosis, interstitial pneumonitis, restrictive lung disease or obstructive lung disease; (4) haematopoietic cell transplant with any history of graft-versus-host disease and the development of pulmonary toxicity, bronchiolitis obliterans, chronic bronchitis or bronchiectasis; and (5) thoracic surgery and the development of pulmonary dysfunction.^22^ However, there may be non-treatment factors that contribute to respiratory mortality. This is particularly apparent for chronic lower respiratory disease deaths in lung cancer survivors, which accounted for 55% (16/29) of the observed excess respiratory deaths in this FPN type, as it is hypothesised that these diseases share underlying host susceptibility factors.^23^ Similarly, the fact that over 60% (46/77) of the pneumonia deaths in childhood cancer survivors occurred in survivors of CNS tumours or CNS PNET suggests an increased risk of aspiration pneumonia, which may be linked to a weaker cough reflex due to chronic neurological deficits.^24^

Another interesting finding from our study is that there were an increasing number of excess respiratory deaths as survivors aged. These findings may relate to the fact that respiratory death is among the more common causes of death among mature members of the general population. Consequently, because the expected number of deaths is substantial, even a modest multiplicative excess risk will result in substantial excess numbers of death.

Nonetheless, it was reassuring that the excess number of respiratory deaths declined among those treated more recently in both cohorts. Such declines could be due to changes in cancer treatments, with the newer treatment regimens designed to reduce radiotherapy and chemotherapy exposures while maintaining long-term, disease-free survival^3 25^; moving forward, continued improvement is a reasonable expectation, especially with the use of proton beam therapy, which will further limit the dose of radiotherapy scatter to the lungs. Other potential contributors include the increased surveillance of late effects through regular follow-up and screening, as well as increased supportive care with improved antiviral and antifungal agents that may limit the severity of lung infections and complications compared with previously.^3 25 26^

### Clinical implications

Although the findings presented in this study provide reliable (based on large numbers) and unbiased (population-based design) evidence on respiratory mortality risks, it is important that these results also inform clinicians and the vulnerable subgroups of survivors identified about the risks of respiratory morbidity. At-risk survivors and survivors with reduced lung reserve/function or abnormal/damaged lung tissues from cancer treatment should be monitored for chronic cough or dyspnoea through yearly pulmonary exams^18 22^ and should be aware of their susceptibility to further insults from recurrent respiratory infections (eg, pneumonia), lifestyle factors (eg, smoking^27 28^ or occupational exposure to toxic fumes/substances^29^) and other medical interventions (eg, exposure to high oxygen concentration with anaesthesia^30–32^); such subsequent insults can lead to further significant deterioration in lung function and reserve, respiratory failure and death. Furthermore, pneumococcal vaccination should be considered an important intervention for childhood, adolescent and young adult cancer survivors as pneumonia was the most common form of respiratory death in all groups, with substantial excess risks observed compared with that expected; although influenza-related deaths were observed less frequently in this study, influenza may act as a precursor to more serious respiratory conditions like pneumonia, and thus influenza vaccination may also be a useful preventative strategy for at-risk survivors. Finally, as the risks of respiratory mortality varied depending on the age at diagnosis of the survivor and the type of death being investigated, clinical follow-up guidelines should be refined in order to provide more accurate suggestions for survivors depending on whether they are a childhood, adolescent or young adult survivor, while interventions relating to specific respiratory complications should be developed.

### Limitations

The first limitation of our study is that we only included individuals who survived at least 5 years after their cancer diagnosis, and thus the risks presented in this study are not representative of the risks for childhood, adolescent and young adult cancer survivors overall. Additionally, our calculations of SMRs and AERs are likely an underestimation of the multiplicative and additive excesses as competing causes of death are more common in our study population than in the general population^33^; as there is no accurate way to adjust for this bias,^33^ we acknowledge that our findings are likely to be conservative. Another limitation is that our study faced data sparsity for some stratifications; despite this being the largest study of respiratory mortality among such survivors, the outcome in question remains rare and thus our results with less than five observed events should be interpreted with caution. Furthermore, due to the fact that we determined respiratory deaths based on the underlying cause of death coded on the death certificate, there is possibly misclassification in our data as the underlying cause of death has imperfect accuracy.^34–36^ This study also lacked detailed treatment information, which prevented any examination of dose response patterns or treatment interactions. Information was also not available on lifestyle factors, making it impossible to assess the relative importance of smoking status, occupational hazards and other environmental factors. Finally, our findings suggest a significant increase in excess respiratory deaths as age increases. Although these findings provide important information for current survivors aged at least 50 years, it will be important to reassess our findings to see whether the risks remain consistent as younger survivors reach this age milestone since the cancer profile of 5 year survivors and treatment have changed over time. Similarly, it will be important to reinvestigate temporal trends in late respiratory mortality risks with additional follow-up in order to determine whether those most recently diagnosed remain with decreased risks.

## Conclusions

This large-scale, population-based study provides evidence that survivors of childhood, adolescent and young adult cancers are at an increased risk of respiratory death, compared with that expected, both overall and for specific types of respiratory death. Although we report appreciable excess risks for respiratory death among specific FPN types and survivors aged over 50 years, there is evidence of a reduction in the excess number of respiratory deaths among individuals more recently diagnosed. These findings substantially advance the current knowledge on respiratory late effects among childhood, adolescent and young adult cancer survivors and will be useful for both survivors and those involved in their clinical management and follow-up.
