# Supplementary material for: Respiratory mortality of childhood, adolescent and young adult cancer survivors
Source: Thorax. 2018 May 10;73(10):959–68. doi: 10.1136/thoraxjnl-2017-210683 (PMC6166601; doi:10.1136/thoraxjnl-2017-210683)
Supplement: Supplementary file 1 [file thoraxjnl-2017-210683supp001.pdf]

Supplemental Material: Respiratory mortality among 235,434 five-year survivors of childhood, adolescent, and young adult cancer

Miranda M Fidler, Raoul C Reulen, Chloe J Bright, Katherine E Henson, Julie S Kelly, Meriel Jenney, Antony Ng, Jeremy Whelan, David L Winter, Clare Frobisher, Michael M Hawkins
On behalf of the British Childhood Cancer Survivor Study (BCCSS) Steering Group

Corresponding author:
Michael Hawkins - telephone: +44 (0) 121-414-7924; fax: +44 (0) 121 4147923; email: m.m.hawkins@bham.ac.uk; address: Centre for Childhood Cancer Survivor Studies, Institute of Applied Health Reseach, University of Birmingham, Birmingham, UK

First Primary Neoplasm Type	ICD-0-3 Histology	ICD-O-3 Site
CNS tumor (excluding PNET)		
Ependymomas and choroid plexus tumor	9383, 9390-9394	C000-C809
Astrocytomas	9380<br>9384, 9400-9411, 9420, 9421-9424, 9440-9442	C723<br>C000-C809
Other gliomas	9380<br>9381, 9382, 9430, 9444, 9450, 9451, 9460	C700-C722, C724-C729, C751, C753<br>C000-C809
Other specified intracranial and intraspinal neoplasms	8270-8281, 8300, 9350-9352, 9360-9362, 9412, 9413, 9492, 9493, 9505-9507, 9530-9539, 9582	C000-C809
Unspecified intracranial and intraspinal neoplasms	8000-8005	C700-C729, C751-C753
CNS PNET	9470-9474, 9480, 9508<br>9501-9504	C000-C809<br>C700-C729
Leukemia (excluding AML)*		
Lymphoid leukemias	9820, 9823, 9826, 9827, 9831-9837, 9940, 9948	C000-C809
Chronic myeloproliferative diseases	9863, 9875, 9876, 9950, 9960-9964	C000-C809
Myelodysplastic syndrome and other myeloproliferative diseases	9945, 9946, 9975, 9980, 9982-9987, 9989	C000-C809
Unspecified and other specified leukemias	9800, 9801, 9805, 9860, 9930	C000-C809
AML	9840, 9861, 9866, 9867, 9870-9874, 9891, 9895-9897, 9910, 9920, 9931	C000-C809
Hodgkin Lymphoma	9650-9655, 9659, 9661-9665, 9667	C000-C809
Non-Hodgkin Lymphoma	9591, 9670, 9671, 9673, 9675, 9678-9680, 9684, 9689-9691, 9695, 9698-9702, 9705, 9708, 9709, 9714, 9716-9719, 9727-9729, 9731-9734, 9760-9762, 9764-9769, 9970	C000-C809
Neuroblastoma	9490, 9500	C000-C809
Non-Heritable Retinoblastoma	9510-9514	C000-C809
Heritable Retinoblastoma	9510-9514	C000-C809
Wilms	8959, 8960, 8964-8967<br>8963, 9364	C000-C809<br>C649

eTable 1: Classification of first primary neoplasms within the British Childhood Cancer Survivor Study

Abbreviations: AML – acute myeloid leukemia; CNS – central nervous system; ICD-O – International Classification of Diseases for Oncology version 3; PNET – primitive neuroectodermal tumor

*referred to in text as ‘non-AML’

First Primary Neoplasm Type	ICD-0-3 Histology	ICD-O-3 Site
Bone Sarcoma		
Osteosarcomas	9180-9187, 9191-9195, 9200	C400-C419, C760-C768, C809
Chondrosarcomas	9210, 9220, 9240	C400-C419, C760-C768, C809
	9221, 9230, 9241-9243	C000-C809
Ewing tumor and related sarcomas of bone	9260	C400-C419, C760-C768, C809
	9363-9365	C400-C419
Other specified malignant bone tumors	8810, 8811, 8823, 8830	C400-C419
	8812, 9250, 9261, 9262, 9270-9275, 9280-9282, 9290, 9300-9302, 9310-9312, 9320-9322, 9330, 9340-9342, 9370-9372	C000-C809
Unspecified malignant bone tumors	8000-8005, 8800, 8801, 8803-8805	C400-C419
Soft Tissue Sarcoma		
Rhabdomyosarcomas	8900-8905, 8910, 8912, 8920, 8991	C000-C809
Fibrosarcomas, peripheral nerve sheath, and other fibrous neoplasms	8810, 8811, 8813-8815, 8821, 8823, 8834-8835	C000-C399, C440-C768, C809
	8820, 8822, 8824-8827, 9150, 9160, 9491, 9540-9571, 9580	C000-C809
Kaposi sarcoma	9140	C000-C809
Other specified soft tissue sarcomas	8587, 8710-8713, 8806, 8831-8833, 8836, 8840-8842, 8850-8858, 8860-8862, 8870, 8880, 8881, 8890-8898, 8921, 8982, 8990, 9040-9044, 9120-9125, 9130-9133, 9135, 9136, 9141, 9142, 9161, 9170-9175, 9231, 9251, 9252, 9373, 9581	C000-C809
	8830	C000-C399, C440-C768, C809
	8963	C000-C639, C659-C699, C739-C768, C809
	9180, 9210, 9220, 9240	C490-C499
	9260	C000-C399, C470-C759
	9364	C000-C399, C470-C639, C659-C699, C739-C768, C809
	9365	C000-C399, C470-C639, C659-C768, C809
Unspecified soft tissue sarcomas	8800-8805	C000-C399, C440-C768, C809
Germ Cell Tumors		
	9060-9065, 9070-9072, 9080-9085, 9100, 9101	C700-C729, C751-C753
	9060-9065, 9070-9072, 9080-9085, 9100-9105	C000-C559, C570-C619, C630-C699, C739-C750, C754-C768, C809
	9060-9065, 9070-9073, 9080-9085, 9090, 9091, 9100, 9101	C569, C620-C629

eTable 1 (continued): Classification of first primary neoplasms within the British Childhood Cancer Survivor Study

Abbreviations: AML – acute myeloid leukemia; CNS – central nervous system; ICD-O – International Classification of Diseases for Oncology version 3; PNET – primitive neuroectodermal tumor

*referred to in text as ‘non-AML’

First Primary Neoplasm Type	ICD-0-3 Histology	ICD-O-3 Site
Breast	8010-8589	C500-C509
Testicular	8101-8239, 8246-8580, 8590-8650, 9060-9105	C620-C629
Cervical	8010-8589	C530-C539
Melanoma	8720-8780	C000-C809
CNS tumor		
Astrocytoma	9380<br>9400, 9401, 9410-9424, 9440-9442	C723<br>C000-C809
Other glioma	9381-9384, 9430, 9444, 9450, 9451, 9460<br>9380	C000-C809<br>C000-C722, C724-C809
Epdenymoma	9391-9394	C000-C809
Medulloblastoma and other PNET	9260, 9364, 9365, 9470-9474	C700-C716
Other specified intracranial and intraspinal neoplasms	9350-9352, 9390, 9530-9539<br>8000-8589<br>8000-8589, 9360-9362<br>8140, 8270-8281, 8300, 9161, 9480, 9493, 9505, 9508, 9540-9571	C000-C809<br>C751, C752<br>C753<br>C700-C729
Unspecified intracranial and intraspinal neoplasms	8000-8004, 8010, 9990	C700-C729
Hodgkin Lymphoma	9650-9667	C000-C809
Non-Hodgkin Lymphoma	9590-9649, 9670-9714, 9716-9719, 9723, 9727-9729, 9755	C000-C809
Thyroid	8010-8589	C739
Gastrointestinal	8010-8589	C150-C218, C220-C269
Soft Tissue Sarcoma		
Fibromatous neoplasms	8810, 8811, 8113-8815, 8830, 8832, 8833, 8835, 8836	C000-C809
Rhabdomyosarcoma	8900-8921, 8991	C000-C809
Other soft tissue sarcoma	8804, 8840, 8850-8881, 8890-8896, 8990, 9014, 9015, 9040-9044, 9120-9160, 9170, 9251, 9252, 9561, 9580-9582<br>9161, 9540-9571	C000-C809<br>C000-C699, C730-C809
Unspecified soft tissue sarcoma	8800-8803, 8805, 8806	C000-C399, C420-C809
Ovary	8010-8589, 8590-8650, 8670, 9000, 9060-9105	C569
Bladder	8010-8589	C670-C679
Other Genitourinary	8010-8589<br>8959-8962	C649, C510-C529, C540-C559, C570-C579, C589, C600-C619, C630-C639, C659, C669, C680-C689<br>C000-C809
Head & Neck	8010-8589	C000-C148, C300-C349, C760
Leukemia (excluding AML)*		
Acute lymphoid leukemia	9826, 9827, 9831, 9832, 9833, 9834-9837, 9948	C000-C809
Chronic myeloid leukemia	9863, 9875, 9876	C000-C809
Other and unspecified leukemia	9733, 9742, 9805, 9820, 9822, 9823, 9830, 9831, 9841, 9842, 9850, 9860, 9862, 9864, 9865, 9867, 9868, 9870, 9880, 9890, 9892-9894, 9900, 9920, 9930, 9931, 9932, 9940, 9941, 9945, 9946, 9963, 9964	C000-C809

eTable 2: Classification of first primary neoplasms within the Teenage and Young Adult Cancer Survivor Study
Abbreviations: AML – acute myeloid leukemia; CNS – central nervous system; ICD-O – International Classification of Diseases for Oncology version 3; PNET – primitive neuroectodermal tumor
*referred to in text as ‘non-AML’

First Primary Neoplasm Type	ICD-0-3 Histology	ICD-O-3 Site
Bone Tumor		
Osteosarcoma	9180-9187, 9192-9195	C000-C809
Chondrosarcoma	9220-9240, 9242, 9243	C000-C809
Ewing tumor	9260, 9364, 9365, 9470-9474	C000-C699, C730-C809
Other specified and unspecified bone tumors	8812, 9250, 9261, 9370-9372<br>8000-8004, 8800, 8801, 8803, 8805, 8806	C000-C809<br>C400-C419
AML	9840,9861, 9866, 9871-9874, 9891, 9910, 9942, 9895, 9896, 9897, 9920	C000-C809
Lung	8010-8589	C330-C349

eTable 2 (continued): Classification of first primary neoplasms within the Teenage and Young Adult Cancer Survivor Study

Abbreviations: AML – acute myeloid leukemia; CNS – central nervous system; ICD-O – International Classification of Diseases for Oncology version 3; PNET – primitive neuroectodermal tumor

*referred to in text as 'non-AML'

International Classification of Diseases (ICD) version	All Respiratory	Pneumonia	Chronic Lower Respiratory Disease	Fibrosis	Pneumonitis	Other Respiratory
**ICD 7**	470-527	480, 490-493	500-502, 526, 527.1	524	525	470-479,481-483, 510-523, 527.0, 527.2
**ICD 8**	460-519	471, 480-486	518, 490-492	NA	517	460-470, 472-474, 493-516, 519
**ICD 9**	460-519	480-486	490-492, 494, 496	495.0, 495.1, 500-505, 506.4, 508.1, 515,516.3	495.7-495.9, 506.0, 507, 508.0, 516.8, 518.3	460-478, 487, 493, 495.2-495.6, 506.1-506.3, 506.9, 508.8-508.9,510-514, 516.0-516.2, 516.9, 517, 518.0-518.2, 518.4-519.9
**ICD 10**	J00-J99	J12-J18	J40-J44, J47	J60.0-J63.5, J66.0, J67.0, J67.1, J68.1, J68.4, J70.0	J67.0, J67.1,J67.7-J67.9, J68.0, J69, J84.8, J95.4	J00-J11, J20-J39, J45-46, J60.0-J63.5, J63.8-J66.0, J66.1-J66.8, J67.2-J67.6, J68.1-J68.4, J68.8-68.9, J70.1-J84.1, J84.9-J95.3, J95.5-J99.8

eTable 3: International Classification of Diseases categorizations and sub-categorizations for respiratory causes-of-death as used in the analysis
Abbreviations: NA – not applicable

Cause-of-Death	Observed/Expected	SMR (95%CI)	AER (95%CI)
All respiratory	164/24.2	6.8 (5.8,7.9)	2.3 (1.8,2.7)
Pneumonia	77/9.4	8.2 (6.5,10.2)	1.1 (0.8,1.4)
Chronic lower respiratory disease	11/6.1	1.8 (0.9,3.2)	0.1 (-0.0,0.2)
Fibrosis	15/1.1	13.8 (7.7,22.8)	0.2 (0.1,0.3)
Pneumonitis	18/1.1	16.9 (10.0,26.8)	0.3 (0.1,0.4)
Other respiratory diseases	43/6.6	6.5 (4.7,8.8)	0.6 (0.4,0.8)

eTable 4: Observed and expected number of deaths, standardized mortality ratios, and absolute excess risks per 10,000 for deaths due to all respiratory causes and respiratory-specific causes within the British Childhood Cancer Survivor Study
Abbreviations: SMR – standardized mortality ratio, AER – absolute excess risk; CI – confidence intervals
